# Area-Level Socioeconomic Disadvantage and Health Care Spending: A Systematic Review

**DOI:** 10.1001/jamanetworkopen.2023.56121

**Published:** 2024-02-15

**Authors:** Anna M. Morenz, Joshua M. Liao, David H. Au, Sophia A. Hayes

**Affiliations:** 1 Department of Medicine, University of Washington, Seattle; 2 Program on Policy Evaluation and Learning in the Pacific Northwest, Seattle, Washington; 3 Now with Department of Medicine, University of Texas Southwestern Medical Center, Dallas; 4 Now with Program on Policy Evaluation and Learning, Dallas, Texas; 5 Center of Innovation for Veteran-Centered and Value-Driven Care, Veterans Affairs Puget Sound Health Care System, Seattle, Washington

## Abstract

**Importance:**

Publicly available, US Census–based composite measures of socioeconomic disadvantage are increasingly being used in a wide range of clinical outcomes and health services research. Area Deprivation Index (ADI) and Social Vulnerability Index (SVI) are 2 of the most commonly used measures. There is also early interest in incorporating area-level measures to create more equitable alternative payment models.

**Objective:**

To review the evidence on the association of ADI and SVI with health care spending, including claims-based spending and patient-reported barriers to care due to cost.

**Evidence Review:**

A systematic search for English-language articles and abstracts was performed in the PubMed, Web of Science, Embase, and Cochrane databases (from inception to March 1, 2023). Peer-reviewed articles and abstracts using a cross-sectional, case-control, or cohort study design and based in the US were identified. Data analysis was performed in March 2023.

**Findings:**

This review included 24 articles and abstracts that used a cross-sectional, case-control, or cohort study design. In 20 of 24 studies (83%), ADI and SVI were associated with increased health care spending. No association was observed in the 4 remaining studies, mostly with smaller sample sizes from single centers. In adjusted models, the increase in spending associated with higher ADI or SVI residence was $574 to $1811 for index surgical hospitalizations, $3003 to $24 075 for 30- and 90-day episodes of care, and $3519 for total annual spending for Medicare beneficiaries. In the studies that explored mechanisms, postoperative complications, readmission risk, and poor primary care access emerged as health care system–related drivers of increased spending.

**Conclusions and Relevance:**

The findings of this systematic review suggest that both ADI and SVI can play important roles in efforts to understand drivers of health care spending and in the design of payment and care delivery programs that capture aspects of social risk. At the health care system level, higher health care spending and poor care access associated with ADI or SVI may represent opportunities to codesign interventions with patients from high ADI or SVI areas to improve access to high-value health care and health promotion more broadly.

## Key Points

**Question:**

Are area-level measures of socioeconomic disadvantage, specifically Area Deprivation Index (ADI) and Social Vulnerability Index (SVI), associated with health care spending?

**Findings:**

In this systematic review of 24 cross-sectional, case-control, and cohort studies, higher ADI and SVI were mostly associated with increased health care spending and patient-reported barriers to care due to cost.

**Meaning:**

Both ADI and SVI may represent important levers to understand drivers of health care spending in the design of payment and health care delivery systems as well as to improve access to high-value care for historically marginalized communities.

## Teaser

This systematic review examines the association of Area Deprivation Index and Social Vulnerability Index with health care spending and patient-reported barriers to care due to cost.

## Introduction

Health and well-being depend in part on access to resources such as transportation, grocery stores, and safe areas for exercise as well as freedom from stressors such as community violence, high rent, and poverty.^1^ Many components of one’s lived environment are shaped by socioeconomic status, which was seminally described as a “fundamental cause” of disease by Link and Phelan^2^ in 1995, given its persistent effect on multiple disease outcomes despite interventions on intermediate mechanisms. Over several decades, composite measures have been developed to capture the presence and extent of socioeconomic disadvantage within geographic areas with more explanatory power than single area measures. Two of the most widely studied and publicly available of these measures are Area Deprivation Index (ADI)^3,4^ and Social Vulnerability Index (SVI).^5^

Both ADI and SVI capture area-level measures of income, employment, education, and household characteristics, including vehicle access, derived from the US Census Bureau American Community Survey. In addition, SVI incorporates measures of population demographics, and ADI includes more measures of poverty and housing costs (Table 1). Whereas ADI is a single composite measure, SVI is available as an overall measure or as 4 submeasures: socioeconomic status, household characteristics, racial and ethnic minority status, and housing type and transportation. State and national ADI is available at the US Census block group level (with the ability to link to a 9-digit zip code), and state and national SVI is available at the US Census tract or county levels. Both ADI and SVI were developed with different intentions: SVI was developed in 2011 by the Centers for Disease Control and Prevention (CDC) to identify communities at greatest need before, during, or after disasters,^5^ whereas ADI was developed by Dr Gopal Singh in 2003 to document the extent of social disparities in health and mortality.^3^

**Table 1.** Comparison of US Census–Based Measures Included in SVI vs ADI

US Census–based socioeconomic measure	SVI (2018)	ADI (2019)
Income and poverty		
Median family income, $	Yes	Yes
Families below federal poverty level, %	Yes	Yes
Population <150% of federal poverty level, %	No	Yes
Income disparity	No	Yes
Employment, %		
Unemployed	Yes	Yes
Employed people aged ≥16 y in white-collar occupation	No	Yes
Education, %		
Population aged ≥25 y with at least high school education	Yes	Yes
Population aged ≥25 y with less than ninth grade education	No	Yes
Population demographic, %		
Aged ≥65 y	Yes	No
Aged ≤17 y	Yes	No
Racial and ethnic minority status^a^	Yes	No
Aged ≥5 y with a disability	Yes	No
Aged ≥5 y and speaks English “less than well”	Yes	No
Household resources and composition, %		
Single-parent household	Yes	Yes
Household without a vehicle	Yes	Yes
Household without a telephone	Yes	Yes
Household with >1 person per room	Yes	Yes
Housing characteristics, %		
Occupied housing units without complete plumbing	No	Yes
Owner-occupied housing units	No	Yes
Housing units with ≥10 units in structures	Yes	No
Housing units that are mobile homes	Yes	No
Persons in institutionalized group quarters (eg, correctional institutions, nursing homes, military quarters)	Yes	No
Housing costs, $		
Weighted median monthly mortgage	No	Yes
Weighted median gross rent	No	Yes
Weighted median home value	No	Yes

Abbreviations: ADI, Area Deprivation Index; SVI, Social Vulnerability Index.

^a^ Defined as American Indian or Alaska Native, Black or African American, Hispanic or Latino, Native Hawaiian and Other Pacific Islander, other race or ethnicity, or multiple races or ethnicities.

Both ADI and SVI have been associated with health outcomes (eg, maternal mortality^6^ and COVID-19 mortality^7^) and with health care utilization measures (eg, readmission risk,^8,9,10^ low acuity emergency department [ED] visits, high ED use, and potentially preventable hospitalizations^11,12^). Currently, ADI is being used in primary care and population-based payment models to calculate benchmarks or payment rates.^13,14^ Studies have pointed to an association between higher area-level socioeconomic disadvantage and health care spending, but this has not been systematically reviewed to discern overall trends or to compare the associations with spending between ADI and SVI. Filling this knowledge gap would enable policy makers to better select and incorporate area-level measures into payment or delivery models and for practice leaders to better engage in these models as well as in opportunities to use these measures to inform the care of their patient populations. This study sought to do so by systematically reviewing the literature on the association between ADI and SVI as area-level measures and health care spending.

## Methods

This systematic review was conducted in accordance with the Preferred Reporting Items for Systematic Reviews and Meta-Analysis (PRISMA) guideline. It was registered with PROSPERO (CRD42023404609).

### Study Inclusion Criteria

We included observational cohort studies, cross-sectional studies, and review articles evaluating the association among ADI, SVI, or both as a primary exposure and health care spending as a primary or secondary outcome. We selected ADI and SVI from among available area-based socioeconomic deprivation indices based on their public availability, accessibility (precreated indices), frequent use in the literature, unique application to the most granular geographic level at the US Census block group (ADI), and ability to subcategorize (SVI).^15^ We included peer-reviewed journal articles and peer-reviewed abstracts from conference proceedings. We excluded editorials, commentaries, and those not subjected to peer review. We included only English-language articles based in the US, given the unique health care environment of the US and study scope. We developed our search strategy using a range of health care spending outcomes, mostly from the payer perspective as follows: total spending; spending for inpatient, outpatient, pharmacy, or postacute care; disease- or episode-specific spending; prospective spending; and 1 outcome focused on patient-borne spending (self-reported spending or barriers to care due to cost) (Box).

Box. **Health Care Spending Outcomes**

- Total spending
- Inpatient spending
- Postacute care spending (including acute rehabilitation, skilled nursing facility, outpatient postdischarge, or postsurgical care)
- Outpatient spending, including primary care spending
- Pharmacy spending
- Prospective or future spending
- Disease- or episode-specific spending
- Self-reported health care spending or barriers to care related to cost

### Identification and Selection of Studies

We searched the PubMed, Web of Science, Embase, and Cochrane databases (from inception to March 1, 2023), developing a search strategy in consultation with a research librarian. Our selection process is demonstrated in the Figure according to the PRISMA flowchart. One author (A.M.M.) performed title, abstract, and full-text screening. Twenty percent of studies were rescreened by a second author (S.A.H.) to verify validity, and any disagreements were resolved through consensus.

**Figure.**
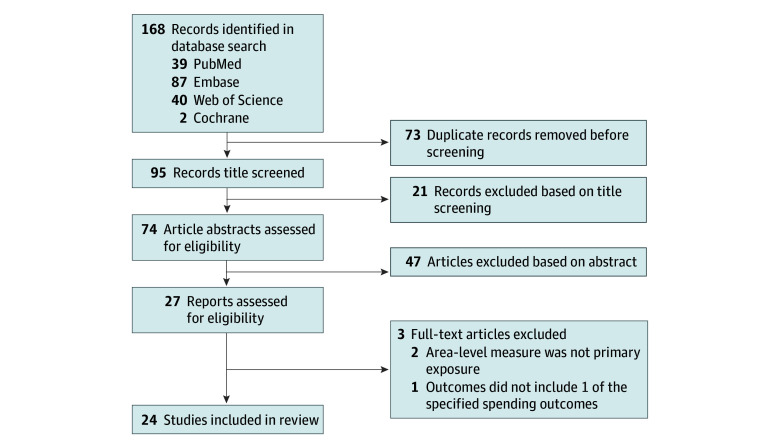
Flow Diagram for Systematic Review Search

### Data Extraction

We created an evidence table and extracted relevant information on specialty area, study design, study population, data source, level and categorization of ADI or SVI exposure, outcomes, statistical analysis, findings, and limitations (eTables 1 and 2 in Supplement 1). One author (A.M.M.) initially extracted this information from each included article, and then data extraction was verified by a second author (S.A.H.) working independently. Data analysis was performed in March 2023, with Excel, version 16.78.3 (Microsoft Corp).

## Results

After 168 articles from 4 peer-reviewed literature registries were screened, 21 were excluded based on the title alone and 47 were excluded based on the abstract. Our search ultimately included 24 unique articles or abstracts (Figure).^12,16,17,18,19,20,21,22,23,24,25,26,27,28,29,30,31,32,33,34,35,36,37,38^

### Study Characteristics

All 24 included studies were published between 2020 and 2023. Characteristics of the included studies are described in Table 2. Slightly over half of the studies (14 [58%]) used ADI and most often at the granular level of the US Census block group or 9-digit zip code cross-walked to US Census block group (13 of 14 [93%]). The remaining studies (10 [42%]) used SVI, which was most often derived from the US Census tract level (n = 2), county level (n = 4), or state level (n = 2). Nearly half of the SVI-based studies (4 of 10 [40%]) included analyses of any SVI submeasures. The SVI-based studies reported using the 2018 (n = 4) or 2020 (n = 1) version of the CDC SVI metric, or they did not indicate the version year (n = 5) but referenced the CDC SVI website^5^ or original 2011 study from Flanagan et al^39^ that informed the CDC SVI. There was heterogeneity in how ADI and SVI were categorized. In some studies, ADI and SVI were binarized at various cut-points; in others, they were categorized by tertiles, quartiles, quintiles, or deciles.

**Table 2.** Selected Characteristics of the 24 Studies Evaluating the Association of ADI or SVI With Health Care Spending

Characteristic	No. (%) of studies		
	With ADI as exposure (n = 14)	With SVI as exposure (n = 10)	Total (N = 24)
Study design			
Retrospective cohort	8 (57)	5 (50)	13 (54)
Retrospective case control	1 (7)	NA	1 (4)
Cross-sectional	3 (21)	4 (40)	7 (29)
Other	2 (14)	1 (10)	3 (13)
Study setting			
Single institution	5 (36)	2 (20)	7 (29)
Multiple institutions	2 (14)	NA	2 (8)
Citywide	1 (7)	1 (10)	2 (8)
Statewide	3 (21)	1 (10)	4 (17)
National	3 (21)	6 (60)	9 (38)
Specialty or patient population			
General adult medicine or overall spending	5 (36)	2 (20)	7 (29)
Surgery (orthopedics)	2 (14)	2 (20)	4 (17)
Surgery (other)	3 (21)	3 (30)	6 (25)
Oncology	3 (21)	NA	3 (13)
Infectious disease or COVID-19	NA	2 (20)	2 (8)
Cardiology	NA	1 (10)	1 (4)
Pediatrics	1 (7)	NA	1 (4)
Geographic level of socioeconomic disadvantage			
US Census block group level (or 9-digit zip code cross-walked to Census block group)	13 (93)	1 (10)	14 (58)
Zip code	NA	1 (10)	1 (4)
US Census tract	NA	2 (20)	2 (8)
County	1 (7)	4 (40)	5 (21)
State	NA	2 (20)	2 (8)
Analytic method			
Multivariable linear regression	7 (50)	6 (60)	13 (54)
Multivariable logistic regression	2 (14)	2 (20)	4 (17)
χ^2^ Test	1 (7)	NA	1 (4)
Least cost path analysis	NA	1 (10)	1 (4)
Kaplan-Meier sample average cost estimator method	1 (7)	NA	1 (4)
Predictive modeling with bootstrapping	1 (7)	NA	1 (4)
Other	2 (14)	1 (10)	3 (13)
Spending outcome			
Spending type			
Hospital profit margins	NA	1 (10)	1 (4)
Total spending	5 (36)	2 (20)	7 (29)
Inpatient hospitalization spending	3 (21)	1 (10)	4 (17)
Potentially preventable inpatient spending	1 (7)	NA	1 (4)
Costs of health care access	NA	1 (10)	1 (4)
Prospective or future spending	1 (7)	NA	1 (4)
Disease- or episode-specific spending	2 (14)	3 (30)	5 (21)
Self-reported health care spending or barriers to care related to cost	2 (14)	2 (20)	4 (17)
Conclusions			
Positive association between ADI or SVI and spending	11 (79)	9 (90)	20 (83)
No association found	3 (21)	1 (10)	4 (17)

Abbreviations: ADI, Area Deprivation Index; NA, not applicable; SVI, Social Vulnerability Index.

Most included studies used a retrospective cohort (13 [54%]) or cross-sectional (7 [29%]) study design. The most common medical specialty areas for these studies included general adult medicine^12,16,17,18,19,20^ (5 of 14 ADI-based studies and 2 of 10 SVI-based studies) or surgery (5 of 14 ADI-based studies and 5 of 10 SVI-based studies), including general,^21,22,23,24^ cardiothoracic,^25^ vascular,^26^ and orthopedic^27,28,29,30^ surgery. A smaller number were more specialty focused on oncology,^31,32,33^ cardiology,^34^ pediatrics,^35^ and COVID-19.^36,37^ Nearly half of the studies using national data (4 of 9 [44%]) involved Medicare claims data. Of the 24 studies, 15 focused more narrowly on data that were from a single institution (7 [29%]; 5 ADI-based and 2 SVI-based), were from multiple institutions (2 [8%]; both ADI-based), were citywide (2 [8%]; 1 ADI-based and 1 SVI-based), or were statewide (4 [17%]; 3 ADI-based and 1 SVI-based). Of these 15 studies, 6 (40%) focused on Medicare beneficiaries.

Over half of the studies (13 of 24 [54%]) used multivariable linear regression to analyze health care spending, adjusting for basic patient sociodemographic variables (eg, age, gender or biological sex, and race and ethnicity), clinical characteristics, and, variably, hospital characteristics. Very few controlled for individual social factors, such as individual education, employment status, or income level, due to limited availability of these variables in electronic medical records and claims data. One study using survey data was able to adjust for individual education, rurality, and marital status.^31^

### Health Care Spending Outcomes

The majority of studies (20 of 24 [83%]) reported a positive association between ADI (11 of 14) or SVI (9 of 10) and at least 1 of the health care spending outcomes included in our search criteria. The most common health care spending outcomes included total spending (7 [29%]; 5 ADI-based and 2 SVI-based) or disease- or episode-specific spending (5 [21%]; 2 ADI-based and 3 SVI-based). Inpatient hospitalization spending was the outcome of interest in 4 of 24 studies (17%; 3 ADI-based and 1 SVI-based). Although postacute care and outpatient spending were often included in total spending and subanalyzed to identify drivers of total spending, no studies solely evaluated these forms of spending as an outcome.

In the ADI-based studies that examined dollar amounts for these spending outcomes from claims data, total spending for surgical care (including postacute care and readmissions) for 4 common surgeries was $2654 higher among those in the highest ADI quintile compared with the lowest,^21^ and index hospitalization spending associated with general surgery cases was $1811 higher for those in the highest ADI quartile.^24^ Overall 90-day spending following a surgical procedure was $3003 to $7415 higher among patients from the highest-decile ADI areas.^25,30^ Total annual spending for Medicare beneficiaries in Maryland was $3519 higher in the highest ADI quintile compared with the lowest^18^ and $48 more per point increase in national ADI percentile for Medicare beneficiaries nationally.^19^ In a study using Medicare fee-for-service claims from New York City–area health systems, residence in the highest ADI quintile was associated with similar total Medicare spending but $53 or 12% higher potentially preventable spending than residence in the middle quintile.^12^ For studies using SVI, spending for an index surgical hospitalization was $574 to $1517 higher for patients from the highest SVI quartile compared with the lowest.^22,23^ For 30-day postdischarge spending following total hip arthroplasty, higher SVI racial and ethnic minority status and language submeasure was associated with $24 075 higher spending.^27^ For 30-day spending after COVID-19 diagnosis, residence in the highest SVI quartile was associated with $3266 higher inpatient and outpatient spending.^37^

Four studies focused on patient-borne spending via self-reported measures of financial toxicity or hardship^31,32^ (ADI-based) or difficulties accessing health care due to cost^16,34^ (SVI-based), with all finding a positive association between increasing area-level disadvantage and these patient-centered outcomes. In 4 studies, spending was a secondary outcome, whereas utilization or quality measures, such as length of stay,^35^ postoperative complications,^22^ 30-day mortality,^24^ and risk of emergent colon operation,^23^ were primary outcomes. No study included a head-to-head comparison of ADI vs SVI. Two SVI-based studies included additional specific measures captured at the area level, specifically residing in a food desert, density of fast-food restaurants, and density of tobacco stores, compared with SVI composite measures.^27,28^

Three single-center studies found no association between ADI or SVI and health care spending.^28,29,33^ One study reported that adding ADI to a claims-based risk adjustment model did not improve prediction of health care spending for commercially insured residents in Maryland.^17^

## Discussion

Based on the available studies, higher ADI and SVI appear to be associated with increased health care spending across a range of patient populations and spending outcomes, from total spending for older patients with chronic diseases to episodic spending following surgery or illness. There is early evidence that these associations may be more pronounced for racial and ethnic minoritized individuals^22^ and individuals with high levels of chronic disease,^18^ but this requires further investigation. In this study, SVI was more frequently used at a larger geographic level (county or state in 6 of 10 SVI-based studies [60%]) than ADI (US Census block group in 13 of 14 ADI-based studies [93%]), which raises concerns about socioeconomic heterogeneity within these larger geographic areas. However, SVI is available at the more granular level of the US Census tract, so this is not necessarily a limitation of SVI but may represent a limitation of other sources of data. In addition, SVI was more commonly used in studies pertaining to COVID-19, potentially due to SVI’s intended purpose to be used in public health emergencies. Interestingly, SVI boasts the advantage of submeasures, but these submeasures were only used in less than half of SVI-based studies, which may reflect either missed opportunities or possibly a concern that submeasures are not amenable to producing valid inference.

Regardless of differences in use and application between ADI and SVI, both were associated with increased health care spending from the payer and patient perspectives. The magnitude of increased health care spending for individuals residing in areas of highest socioeconomic disadvantage ranged widely in adjusted models from $574 to $24 075, partly due to varying definitions for episodes of care. Nonetheless, these increases in health care spending associated with higher neighborhood socioeconomic disadvantage are substantial, particularly when many of the surgical procedures examined (eg, coronary artery bypass grafting, colectomies, and joint replacements) are commonly performed in the US. Although studies examined patient financial hardship and reported barriers to care due to cost, no study quantitatively examined the association between neighborhood socioeconomic disadvantage and health care costs borne by patients, such as insurance premiums and copays.

The mechanism for the positive association between ADI or SVI and health care spending found by most studies was explored in greater depth inconsistently between studies. For studies that found increased episodic spending following a diagnosis or acute surgery, ADI and SVI were often associated with increased lengths of stay, postoperative complications, readmissions, and postacute care spending. This finding could signal a benefit for certain types of alternative payment models, such as bundled payments for surgeries, to incorporate consideration of area-level measures to avoid “lemon dropping” (avoiding care of patients deemed to be at higher risk for complications and high costs), as well as interventions targeting transitions of care for individuals from high ADI or SVI areas. Higher state-level SVI was also associated with individuals reporting the absence of a primary care clinician and an inability to see a doctor because of cost. This lack of access could drive future health care spending due to a lack of preventive care and diagnostic services, leading to greater risk of decompensated illness or advanced presentations of disease.^16,34^ Indeed, one study found that Medicare patients from the highest quintile of ADI had higher preventable spending but lower total spending compared with patients in the lowest quintile, which may indicate unmet needs for necessary health care among these patients.^12^

These potential mechanisms of increased readmission risk or lack of primary care access inadequately capture the full complexity of the association between ADI or SVI and health care spending. Health care spending—and its antecedent, health care utilization—are driven by a complex interplay of multiple factors beyond the traditional reach of the health care system. These associations include not only an individual’s lived environment but also sociopolitical factors (eg, immigration status and institutional racism) and sociocultural factors (eg, social networks and family values surrounding health).^40^ Furthermore, the fact that the positive association between ADI or SVI and health care spending persists across multiple settings and disease outcomes suggests that one’s lived environment may be a “fundamental cause” of health care spending, although, importantly, none of these observational studies can determine causality.^2^ Regardless, as a result of this association, health care systems are faced with the challenge of how best to assess and interface with patients’ complex lived environments.^41^

Findings from this systematic review suggest that ADI and SVI can play important roles (1) in efforts to understand drivers of health care spending and (2) in the design of payment and care delivery programs that capture aspects of social risk. For example, policies and programs could be designed to provide more resources to health care delivery organizations caring for more historically marginalized populations residing in areas with greater social risk. Health care organizations, in turn, could use information from area-level measures to screen their populations for social needs, connect individuals with identified needs to more intensive care management and community resources, and target initiatives and funding to meet community needs.

Our findings suggest that to achieve this goal, policy makers must also address a number of issues. First, they must decide whether to use area-level measures as either composite or more granular measures to achieve policy goals. The aggregative nature of ADI and SVI creates challenges in identifying specific policy and delivery interventions. For instance, it may difficult to identify whether high ADI or SVI reflects limited transportation access, poverty, employment barriers, lack of education, or other aspects of social risk. To that point, one study in this review did not observe an association between SVI and post–joint replacement episodic spending but did observe associations between more specific area-level measures (density of tobacco stores and presence of food deserts) and spending.^28^ Although SVI may offer an advantage in its ability to be decomposed into 4 submeasures, it may nevertheless be limited in what aspects of social risk and lived environment are captured. Concerns have also been raised that substantial inequities can exist within certain areas with low ADI, such as urban areas with high median home values, suggesting the importance of using state rather than national ADI or supplementing with other measures of living costs.^42^ Lack of standardization of dollar amounts (for median rent, mortgage, and home value) in ADI may also underestimate deprivation even when other variables in ADI indicate high deprivation.^43^

Second, policy makers would benefit from more granular understanding of edge cases or exceptions—situations in which area-level measures are not associated with greater spending. This may occur if underlying studies involve narrow data or scope. For example, in this review, 3 of the 4 studies that did not find an association between ADI or SVI and spending were single-center studies with relatively small sample sizes.^28,29,33^ Two of these studies evaluated elective joint replacement surgery, which may not reflect other types of care. Alternatively, these studies may reflect either particularly resilient health care systems or skew in the local-level ADI or SVI. The fourth negative study used statewide commercial claims and concluded that ADI did not improve predictive model performance.^17^ Many of the other studies included in this review used Medicare data, which may differ from commercial populations in terms of medical complexity, health care needs, and susceptibility to other aspects of socioeconomic disadvantage. Ultimately, these examples underscore the need for more work elucidating the specific mechanisms relating area-level measures and health care spending. Predictive modeling of health care spending was also only undertaken in this single study,^17^ and this area merits further investigation.

Third, policy makers should ensure that use of area-level measures is informed by equity considerations. Policy interest and precedence using these measures is growing, particularly given evidence that prior policies have disproportionately penalized safety-net organizations caring for patients with high levels of social needs.^44,45^ For example, ADI has been incorporated into payment adjustment for the recent Accountable Care Organization Realizing Equity, Access, and Community Health model^13^ and the Making Care Primary^46^ model. However, deeper, systemic issues also exist. For example, some patterns of neighborhood resources in the US have been shaped by racist policies, such as redlining, which has disproportionately impacted racial and ethnic minoritized communities.^47^ As feasible, such dynamics could inform the cocreation of more meaningful, restorative partnerships between health care and communities with high ADI. More specifically, health care leaders should actively include local community voices in the development of interventions based on these area-level measures. Appropriate solutions are likely to be highly contextualized and may be structured differently for 2 communities with the same numerical area-level disadvantage. In addition, payment and care delivery models using ADI or SVI should be closely monitored for impacts on historically marginalized groups, given that ADI and SVI may be a signal for individual risk but should not be used as a replacement due to ecological fallacy and evidence that area-level measures are inconsistent indicators of individual-level social risks.^48^

### Limitations

This analysis has several limitations. First, studies included in this review were observational in nature and susceptible to residual confounding. Most studies attempted to address this issue with multivariable regression analysis, and 1 study used propensity score matching. Among the multivariable regression analyses, there were varying levels of adjustment for individual sociodemographic variables and variables such as education, insurance type, dual eligibility status, marital status, or rural residence were used infrequently. Incorporation of individual social variables alongside ADI and SVI to distinguish between area-level vs individual-level effects of social risk should be a priority for future work.

Second, conclusions about ADI and SVI may not be generalizable to certain populations. In particular, no study focused exclusively on Medicaid beneficiaries, who disproportionately experience high social risks. Instead, the majority of studies used Medicare claims data or institutional data linked to claims, which included some Medicaid patients. Further research is needed to explore area-level measures of social risk and their association with health care spending for Medicaid populations.

Third, multiple approaches were used to categorize ADI or SVI for analysis. Future work should elucidate best practices for categorization. Fourth, among the included studies, there was some variation in the geographic level at which analyses were conducted. Such variation should inform interpretation and prompt future study. For instance, findings reported at a more granular US Census block group level, which may be more relevant to neighborhood-level interventions, should be distinguished from findings reported at a larger, more geographically heterogenous county level, which may be more relevant to state or national policy design.

## Conclusions

The findings of this systematic review suggest that the area-level measures ADI and SVI can both be associated with increased health care spending, with most evidence analyzing spending from the payer perspective, but exceptions and a number of outstanding issues exist. More work on this topic is needed, but health care leaders and policy makers can consider policy-, community-, health care system–, and patient-level interventions that incorporate area-level measures to address differential spending levels, support use of cost-effective, high-value care, and advance community-based solutions beyond the walls of the health care system, with close attention to evaluating health outcomes and impacts on equity.

## Notes

Supplement 1. **eTable 1.** Critical Literature Review Matrix for Studies Using Area Deprivation Index **eTable 2.** Critical Literature Review Matrix for Studies Using Social Vulnerability Index

Supplement 2.
Data Sharing Statement
