# Supplementary material for: Area-Level Socioeconomic Disadvantage and Health Care Spending: A Systematic Review
Source: JAMA Netw Open. 2024 Feb 15;7(2):e2356121. doi: 10.1001/jamanetworkopen.2023.56121 (PMC10870184; doi:10.1001/jamanetworkopen.2023.56121)
Supplement: Supplement 1. — eTable 1. Critical Literature Review Matrix for Studies Using Area Deprivation Index eTable 2. Critical Literature Review Matrix for Studies Using Social Vulnerability Index [file jamanetwopen-e2356121-s001.pdf]

Supplementary Online Content

Morenz AM, Liao JM, Au DH, Hayes SA. Area-level socioeconomic disadvantage and health care spending: a systematic review. *JAMA Netw Open*. 2024;7(2):e2356121. doi:10.1001/jamanetworkopen.2023.56121

eTable 1. Critical Literature Review Matrix for Studies Using Area Deprivation Index

eTable 2. Critical Literature Review Matrix for Studies Using Social Vulnerability Index

This supplementary material has been provided by the authors to give readers additional information about their work.

© 2024 Morenz AM et al. *JAMA Network Open.*

eTable 1. Critical Literature Review Matrix for Studies Using Area Deprivation Index

Author	Year	Title	Topic Area	Study Design	Study Population	Data Source	Exposure	Outcome(s)	Statistical Analysis	Findings	Limitations
Bonner, SN	2022	Neighborhood Deprivation and Medicare Expenditures for Common Surgical Procedures	Surgery	Retrospective cohort	Medicare beneficiaries, age 65-99, undergoing appendectomy, colectomy, hernia repair, or cholecystectomy between 2014-2018	Medicare Provider Analysis and Review files, American Hospital Association Annual Survey	Area Deprivation Index (ADI) from 9-digit zip code by national quintile	Total episode of surgical care (index hospitalization, physician fees, post-acute care, and readmission)	Multivariable regression including patient age, gender, race, Elixhauser comorbidities, dual-eligibility status, admission type (elective vs unplanned), year, procedure and hospital characteristics including size, geographic region, teaching status, and patient to nurse ratio; robust standard errors applied to account for clustering within hospitals	Total surgical spending was $2654 higher among beneficiaries from the most deprived neighborhoods compared to the least; these differences were driven by higher readmission rates (12.9% vs 10.8%) and post-acute care (67.8% vs 61.2%)	
Chang, HY	2021	Impact of Area Deprivation Index on the Performance of Claims-Based Risk-Adjustment Models in Predicting Health Care Costs and Utilization	General health care	Predictive modeling	1.15 million Maryland residents aged 18-63 with >=6 months enrollment in 2013-2014	Maryland Medical Care Database (statewide commercial claims database)	National ADI percentile as continuous variable and by deciles	Addition of ADI to predictive models of concurrent and prospective costs (total, pharmacy, medical) and utilization (being top 5% user, having any hospitalization, having any Emergency Department (ED) visit, having any avoidable ED visit, having any readmission)	Linear regression for cost outcomes; R(2) and mean absolute prediction error were compared between the base and the ADI-enhanced models; logistic regression for utilization outcomes; compared AUC and Akike's Information Criterion between the base and ADI-enhanced models; all performance measures and 95% CIs were derived from the bootstrapping analysis with 200 iterations; control variables: age, sex, 1 morbidity measure	Adding ADI did not improve model performance except in predicting the probability of having any ED or any avoidable ED visit	

© 2024 Morenz AM et al. *JAMA Network Open.*

Corkum, J	2022	Area Deprivation Index and Rurality in Relation to Financial Toxicity among Breast Cancer Surgical Patients: Retrospective Cross-Sectional Study of Geospatial Differences in Risk Profiles	Cancer	Cross-sectional	Survey data from a single institution collected between 1/2018-6/2019 representing 568 surgical breast cancer patients (pts)	Electronic medical record (EMR) data	County ADI by tertile of national percentile	Odds of financial toxicity (FT) as identified by the validated Comprehensive Score for Financial Toxicity questionnaire	Multivariable regression adjusted for age, race/ethnicity, cancer stage, education, rurality, insurance type, marital status, reconstruction status, radiation/ chemotherapy, complication within 90 days, and type of cancer surgery	Higher ADI was a/w worsening odds of FT (odds ratio [OR] 1.012, 1.01-1.02)	28.2% response rate to their survey on FT
Fliegner, M	2022	Area Deprivation and Medicare Spending for Coronary Artery Bypass Grafting: Insights From Michigan	Surgery (cardio-thoracic)	Retrospective cohort	8728 Medicare Fee-for-Service (FFS) pts who underwent CABG procedures in Michigan from 1/1/2012-12/31/2018	Michigan Value Collaborative claims data, Michigan Society of Thoracic and Cardiovascular Surgeons Quality Collaborative data	Zip code-based ADI by decile	90-day episode spending after CABG as well as component spending categories for index hospitalization, professional services, post-acute care, and readmissions	Multivariable logistic and linear regression models, adjusting for patient sex, age, race, clinical characteristics	Pts in top ADI decile had higher overall 90-day spending ($3003 higher, p=0.001). Spending was higher for index hospitalizations, professional services, and readmissions. Inpatient rehab was the only significant difference in post-acute care spending (not SNF, home health, or outpatient facility).	

© 2024 Morenz AM et al. *JAMA Network Open.*

Gordon, AM	2023	Lower Neighborhood Socioeconomic Status May Influence Medical Complications, Emergency Department Utilization, and Costs of Care After 1-2 Level Lumbar Fusion	Surgery (orthopedics)	Retrospective case-control	34,442 pts in total undergoing primary 1-2 lumbar fusion	Retrospective query of the 2010-2020 PearlDiver database	Zip-code based ADI binarized at 90th percentile	90-day complications, ED utilizations, readmissions, and costs of care	Multivariable linear regression utilizing propensity-score matching based on age, gender, and Elixhauser Comorbidity Index	High ADI pts experienced higher rates and odds of ED visits within 90 days (9.67% vs 8.91%, p=0.014), day of surgery costs ($49,878 vs $42,886, p<0.001) and overall 90-day expenditures ($54,459 vs $47,044; p<0.001). High ADI pts incurred higher rates and odds of developing respiratory failure.	
Liao, K	2021	The Impact of Socioeconomic Disadvantage on Pediatric Tracheostomy Outcomes	Pediatrics (ENT)	Retrospective case series	239 pediatric pts who underwent tracheostomy	Institutional EMR	9-digit zip code-based ADI; divided into less (0-50) and more (51-100) disadvantaged	Primary outcome: total length of stay; secondary outcomes included total hospitalization costs, readmissions, mortality, and quality of life scores	Multi-level mixed effects parametric survival for total length of stay (LOS); analysis of variance for continuous variables between more vs less disadvantaged	More disadvantaged children had significantly higher total hospitalization costs (461k vs 279k, p=0.01) and lengths of stay; similar readmission rates, mortality, and quality of care scores	
Michaels, AD	2021	Socioeconomic risk-adjustment with the Area Deprivation Index predicts surgical morbidity and cost	Surgery	Retrospective cohort	All general surgery cases performed at a single institution from 2005-2015 (n=9483)	Institutional American College of Surgeons National Surgical Quality Improvement Program (ACS NSQIP) database and	Zip code-based ADI by national quartile	Primary outcome: 30-day mortality; secondary outcomes: ACS NSQIP complications, rate of reoperation, duration of stay, 30-day readmission, long-term mortality, and cost	Multivariable logistic regression adjusted for ACS NSQIP Risk Score; secondary outcomes analyzed using Kaplan-Meier and Cox proportional hazards models and inflation-adjusted hospital cost using linear regression modeling	ADI independently predicted major postoperative complications (OR 1.11, p=0.02), improved the discrimination of risk-stratification	

© 2024 Morenz AM et al. *JAMA Network Open.*

						institutional Clinical Data Repository				when added to the NSQIP calculator (AUC 0.758-0.790, p=0.02), and was a/w hospitalization cost ($1811/quartile, p=0.03). ADI not a/w 30-d readmission, 30-d mortality, or long-term survival.	
Moverman, MA	2022	Neighborhood socioeconomic disadvantage does not predict outcomes or cost after elective shoulder arthroplasty	Surgery (orthopedics)	Retrospective cohort	380 pts undergoing elective total shoulder arthroplasty from 2015-2018 in their institutional registry	Prospectively maintained institutional registry	Census-block group state ADI grouped into least disadvantaged (1-3), middle (4-6), and most (7-10)	Hospitalization time-driven activity-based costs and 2-year post-operative American Shoulder and Elbow Surgeons score and pain intensity score	Bivariate analyses using Pearson Chi-squared test for categorical variables and independent-samples *t* test for continuous variables; actual dollar amounts not reported so pts categorized into top quartile of total in-hospital costs vs lower 3 quartiles	No differences in hospitalization costs by ADI group; most disadvantaged group had similar pre-op and post-op and pain intensity scores compared to other ADI groups	Multivariable regression not performed but baseline characteristics overall very similar between the three ADI groups; time-driven activity-based costing used to estimate costs as individual hospital cost data were confidential
Sapra, KJ	2020	Identifying High-Cost Medicare Beneficiaries: Impact of Neighborhood Socioeconomic Disadvantage	General health care	Observational longitudinal study	Observational longitudinal study of Maryland FFS Medicare beneficiaries from 2015-2016 (n=615,637)	Centers for Medicare & Medicaid Services (CMS) Chronic Condition Warehouse Master Beneficiary File	9-digit zip ADI by quintiles	Primary: total cost of care (TCOC) calculated for 2016; secondary: utilization measures (inpatient LOS, hospital inpatient care, ED visits, physician office visits, and outpatient & ancillary claims) for 3 key ambulatory care sensitive conditions (ACSCs) (diabetes,	Ordinary least squares regression controlling for baseline race/ethnicity, % of spending by teaching hospitals, beneficiary clinical risk measured by HCC score	Highest ADI quintile pts vs lowest ADI incur greater costs in the subsequent year ($12,439 vs $8,920, p<0.001); clinical risk exacerbates this disparity: among beneficiaries in	May be state-specific findings; authors performed exploratory descriptive analysis of national Medicare 2015 claims linked to ADI and found that

© 2024 Morenz AM et al. *JAMA Network Open.*

								COPD/asthma, and heart failure)		the highest HCC score quintile, costs are 24% higher in the highest vs lowest ADI quintile with risk adjustment; healthcare utilization for 3 ACSCs was greater with higher ADI for inpatient, ED, and outpatient visits	many states show correlation between ADI and TCOC, other states showed no such relationship
Shankaran, V	2021	Clinical Characteristics, Treatment Patterns, and Healthcare Costs and Utilization for Hepatocellular Carcinoma (HCC) Patients Treated at a Large Referral Center in Washington State 2007-2018	Cancer	Retrospective cohort	215 pts diagnosed with HCC from 2007-2018	Institutional EMR data linked to cancer registry data and health claims	Zip code-based ADI in two groups (0-5, 6-10)	Survival; treatment and healthcare utilization; healthcare costs (mean lifetime costs from diagnosis to death or end of follow-up)	Multivariate Cox proportional hazards model to investigate factors a/w survival; cost estimates performed with Kaplan-Meier sample average cost estimator method	Survival significantly lower in higher ADI pts (OR 1.57, 1.02-2.4); mean lifetime costs highest in pts with BCLC A disease, attributable to surgery and hospital costs	Pts lacking health insurance or insured by a payer outside their linkage not included (their linkage is with Premera Blue Cross, Regence Blue Shield, Medicare, Medicaid)
Skinner, L	2022	Rethinking Rurality: Using Hospital Referral Regions to Investigate Rural-Urban Health Outcomes	General health care	Cross-sectional	Medicare FFS beneficiaries aged 65-99 and living in 2015; 306 hospital referral regions	The Dartmouth Atlas	Merged Rural-Urban Commuting Area-zip code, hospital referral region-zip code, and national ADI-zip code data sets	Mortality rates, Medicare reimbursements, percent Medicare enrollees who have at least one visit to a primary care physician, diabetic hemoglobin A1c testing rates, and mammography rates.	Linear correlation between rural proportion and ADI using Spearman's rank correlation coefficient; multivariable linear regression adjusted for age, sex, and race	ADI was positively a/w price-adjusted Medicare reimbursement and greater spending, as well as increasing adjusted mortality and % Medicare enrollees who receive an annual primary care visit	

© 2024 Morenz AM et al. *JAMA Network Open.*

Voleti, SS	2021	Financial Hardship Amongst Patients with Hematologic Malignancies: Using the EMR to Streamline and Prioritize Patient-Centered Care (Abstract)	Cancer	Cross-sectional	10, 024 pts from 2018-2020 receiving care for hematologic cancer from 2018-2020	Mayo Clinic Cancer Registry and EMR	Zip-coded based ADI percentile by quintiles	Financial hardship (FH) self-report	Multivariable logistic regression adjusting for age, marital status, race and ethnicity, insurance type, employment, rurality, type of hematologic cancer, and distance from cancer center	Endorsing significantly more FH was more likely in the these highest vs lowest ADI quintile	
Zhang, Y	2020	Association Between Residential Neighborhood Social Conditions and Health Care Utilization and Costs	General health care	Retrospective cohort	93,429 pts enrolled in Medicare FFS or dual eligibles from 2013-2014	6 health systems in New York City	9-digit zip ADI quintile	Total annual Medicare cost of care + preventable ED visits, preventable hospitalizations, and unplanned 30-day hospital readmissions	Generalized linear models and logistic regressions, adjusted for demographic (age, sex, race, dual eligibility) and clinical (comorbidities, end-stage renal disease, frailty) characteristics of each year	Pts from highest ADI quintile had higher preventable costs but lower total Medicare costs	
Zhang, Y	2022	Developing an Actionable Patient Taxonomy to Understand and Characterize High-Cost Medicare Patients	General health care	Retrospective cohort	428,024 Medicare FFS pts in the New York metropolitan area	Medicare FFS claims	Zip-code based ADI; binarized at 70th percentile	% of high-cost patient captured by each category and likelihood that a patient in a given category would be high cost	Descriptive	Slightly higher proportion of high-cost pts had vulnerable social conditions relative to the general patient population	

*Abbreviations *by alphabetical order*:
ACS NSQIP = American College of Surgeons National Surgical Quality Improvement Program, ACSC = ambulatory care sensitive condition, ADI = Area Deprivation Index, BCLC = Barcelona Clinic Liver Cancer staging system, CABG = coronary artery bypass graft, ED = Emergency Department, EMR = electronic medical record, FFS = fee-for-service, FH = financial hardship, FT = financial toxicity, HCC = hepatocellular carcinoma, LOS = length of stay, OR = Odds Ratio, pts = patients, SDOH = social determinants of health, TCOC = total costs of care

© 2024 Morenz AM et al. *JAMA Network Open.*

eTable 2. Critical Literature Review Matrix for Studies Using sSocial Vulnerability Index

Author	Year	Title	Topic Area	Study Design	Study Population	Data Source	Exposure	Outcome(s)	Statistical Analysis	Findings	Limitations
Al Rifai, M	2022	State-Level Social Vulnerability Index and Healthcare Access: The Behavioral Risk Factor Surveillance System Survey	General health care	Cross-sectional	U.S. adults aged >=18 years surveyed from 2016-2019	Behavioral Risk Factor Surveillance System (BRFSS)	State-level Social Vulnerability Index (SVI) by tertiles	Self-reported absence of healthcare coverage, absence of primary care provider, >1 year duration since last routine check-up, inability to see doctor because of cost, composite variable of any difficult accessing health care because of cost	Multivariable logistic regression adjusted for age, gender, race/ethnicity, comorbidity burden, Medicaid expansion state, and rural residence	State-level SVI associated with (a/w) all measures of health care access in adjusted models, including inability to see doctor because of cost (odds ratio [OR] 1.38, 1.23-1.54)	SVI evaluated at state level
Delanois, RE	2022	Social Determinants of Health in Total Hip Arthroplasty: Are They Associated With Costs, Lengths of Stay, and Patient Reported Outcomes?	Surgery (orthopedics)	Retrospective cohort	136 Medicare patients (pts) with complete social determinants of health (SDOH) data who underwent primary total hip arthroplasty (THA) at Sinai Hospital of Baltimore between 2018-2019	Chesapeake Regional Information System for our Patients, American Fact-Finder from the US Census Bureau, Food Access Research Atlas from the USDA, institution's EMR	Census tract SVI (continuous)	30-day post-discharge costs of care, length of stay, and patient-reported outcomes	Multivariable linear regression adjusting for patient demographics, baseline comorbidities, SVI sub-categories, and SDOH factors (tobacco stores, food deserts)	SVI minority status was a/w higher 30-day costs ($24,075 +/- 9845, p=.01), as well as residing in a food desert. No SDOH in the study were a/w length of stay (LOS) or patient-reported outcomes	Small N, single center study
Delanois, RE	2021	Social Determinants of Health in Total Knee Arthroplasty	Surgery (orthopedics)	Retrospective cohort	234 Medicare pts with complete SDOH data who underwent total knee arthroplasty (TKA) at Sinai Hospital of Baltimore between 1/1/2018 - 12/31/2019	Chesapeake Regional Information System for our Patients, US Census Bureau, Food Access Research Atlas from the USDA, institution's EMR	Census tract SVI by 4 sub-categories	30-day length of stay (LOS) and total cost of care (TCOC) after undergoing TKA	Multivariable linear regression adjusting for age, gender, race, marital status, lives alone or not, primary procedure, health status/comorbidities, food access (food desert, convenience store density, fast food restaurant density), and healthcare access (tobacco store density,	SVI sub-categories were not significantly a/w 30-day LOS or TCOC; areas with higher density of tobacco stores were a/w increased LOS and TCOC; food deserts were also a/w	Small N, single center study

© 2024 Morenz AM et al. *JAMA Network Open.*

									pharmacy density, fitness center density)	increased TCOC	
Diaz, A	2021	County-level Social Vulnerability is Associated With Worse Surgical Outcomes Especially Among Minority Patients	Surgery	Retrospective cohort	Medicare beneficiaries who underwent colon resection, coronary artery bypass graft surgery (CABG), lung resection, or lower extremity joint replacement (LEJR) from 2016-2017 (n=299,583)	Medicare Provider Analysis and Review files	County-level SVI by quartile	Probability of post-operative complications (primary outcome) + mortality, readmission, and expenditures a/w index surgical hospitalization	Multivariable, mixed-effects regression models with a random effect for county were used, controlled for age, sex, race, Elixhauser comorbidities, and hospital teaching status; models including an interaction effect between race and SVI were utilized to measure differential effect of racial minority status and SVI	Risk-adjusted expenditures highest among pts from the highest SVI quartiles by ~$2k (p<0.001); incremental differences in expenditures were highest among Black/minority pts from high SVI counties vs White patient from low SVI counties.	Sample in this study was largely White (91%)
Diaz, A	2020	Association of County-Level Social Vulnerability with Elective Versus Non-elective Colorectal Surgery	Surgery (colorectal)	Retrospective cohort	Medicare beneficiaries aged 65-99 who underwent colon resection for diverticulitis (n=11,812) or colon cancer (n=33,312) from 2016-2017	Medicare Provider Analysis and Review files	County-level SVI by quartile	Risk of emergent colon operation (primary outcome); post-operative complications, mortality, readmission, and index hospital expenditure (secondary outcomes)	Multivariable models controlling for age, sex, race, Elixhauser comorbidities, and hospital teaching status	High SVI pts had higher index hospitalization expenditures.	Could not control for severity of diverticular disease or cancer staging (to see if high SVI pts present with more advanced disease)
Puro, N	2021	Community Social Vulnerability Index and Hospital Financial Performance	Health care management	Cross-sectional	3,664 non-federal acute care hospitals in the U.S.	American Hospital Association; Medicare cost reports; Area Health Resource File	County SVI, by quartile	Hospital operating margin and excess margin	Multivariable regression, adjusted for Hirfindahl-Hirschmann index (measure of health care competition in the market), % of pts without insurance, staffed hospital beds, system membership, teaching status, critical access hospital, rural, occupancy rate, Medicare payor mix, Medicaid payor mix,	Hospitals in the highest SVI quartile were a/w lower operating and excess profit margins	

© 2024 Morenz AM et al. *JAMA Network Open.*

									non-profit status, for-profit status, non-federal government hospitals		
Roy, A	2022	Variation in COVID-19 Treatment Cost Due to Social Factors in the US (Abstract)	Infectious disease (COVID-19)	Retrospective cohort	306,652 pts diagnosed with COVID-19 in the U.S. from 2020-2021	U.S. insurance claims database (Optum)	Minority health SVI subtheme by quartiles; zip code-based	Estimated medical cost post-index COVID-19 infection	Not specified	Four times the medical costs for pts in the highest quartile SVI vs lowest ($4374 vs $1108)	
Roy, A	2022	A multicriteria decision analysis framework to measure equitable healthcare access during COVID-19	Infectious disease (COVID-19)	Geo-spatial analysis	Least cost path (LCP) analysis to quantify the costs a/w health care access from each census block group in the Los Angeles (LA) Metro area to the nearest hospital (n=4263)	LA County Public Health Dept, Centers for Disease Control and Prevention, LA County Location Management System, US Census Bureau, LA Dept of Transportation	Census block group SVI by quintiles	Overall accessibility to the nearest healthcare facility for each census block group (measured by the least cost path from the centroid of the census block group to the healthcare facility)	Multi Criteria Decision Analysis and least cost path analysis; in addition to SVI, demographic, economic, and built environment characteristics also included (slope of the street, car ownership, population density distribution, walkability, traffic collision density, and speed limit)	Census block groups with high SVI (>0.75) had low accessibility due to higher costs of access to nearby hospitals; these were also coincident with hotspots for COVID-19 cases and deaths	
Shah, KB	2022	County-Level Social Vulnerability Is Associated With Higher Rates of Vascular Amputations in Florida (Abstract)	Surgery (vascular)	Cross-sectional	38,488 hospitalizations for peripheral artery disease in Florida from 10/2015 - 12/2019	Florida State Inpatient Database	County-level SVI by quartile and by SVI subthemes	Rates of major or minor amputations, in-hospital mortality, 60-day readmissions, total hospital charges	Multivariable regression models, adjusted for patient age, race, and emergent or urgent hospitalizations	Higher rates of amputation in the highest vs lowest SVI quartiles (OR 1.30, 1.11-1.53); worse socioeonomic SVI a/w longer hospital lengths of stay and higher costs but not in-hospital mortality or readmissions	
Thompson, ZM	2022	State-Level Social Vulnerability Index and Healthcare Access in	Cardiology	Cross-sectional	Individuals with ASCVD (n=203,347)	BRFSS data from 2016-2019	State-level SVI by tertiles	Healthcare coverage, presence of primary care clinician, duration since last routine check-up, delay in access to healthcare,	Multivariable logistic regression adjusted for age, gender, race/ethnicity, Medicaid expansion	Those in highest tertile SVI states more likely to report absence of a primary	SVI evaluated at state level

© 2024 Morenz AM et al. *JAMA Network Open.*

		Patients With Atherosclerotic Cardiovascular Disease (from the BRFSS Survey)						inability to see doctor because of cost, cost-related medication non-adherence	state, and rural residence	care clinician (OR 1.33, 1.12-1.58), delay in access to healthcare (1.39, 1.18-1.63), and inability to see a doctor because of cost (1.21, 1.06-1.40)	

*Abbreviations *by alphabetical order*:
ASCVD = atherosclerotic cardiovascular disease, a/w = associated with, BRFSS = Behavioral Risk Factor Surveillance System, CABG = coronary artery bypass graft, ED = Emergency Department, EMR = electronic medical record, LEJR = lower extremity joint replacement, LA = Los Angeles, LOS = length of stay, OR = Odds Ratio, pts = patients, SDOH = social determinants of health, SVI = Social Vulnerability Index, TCOC = total costs of care, THA = total hip arthroplasty, TKA = total knee arthroplasty

© 2024 Morenz AM et al. *JAMA Network Open.*
