# Supplementary material for: Area-Level Socioeconomic Disadvantage and Health Care Spending: A Systematic Review
Source: JAMA Netw Open. 2024 Feb 15;7(2):e2356121. doi: 10.1001/jamanetworkopen.2023.56121 (PMC10870184; doi:10.1001/jamanetworkopen.2023.56121)
Supplement: Supplement 2. — Data Sharing Statement [file jamanetwopen-e2356121-s002.pdf]

Data Sharing Statement

Morenz. Area-Level Socioeconomic Disadvantage and Health Care Spending. *JAMA Netw Open*. Published February 15, 2024. doi:10.1001/jamanetworkopen.2023.56121

Data
Data available: Yes
Data types: Data (not involving human participants)
How to access data: Our systematic review table used for data collection will be made available within our manuscript (as supplemental Table S1).
When available: With publication

Supporting Documents
Document types: None

Additional Information
Who can access the data: As a supplemental table.
Types of analyses: Systematic review table.
Mechanisms of data availability: With investigator support.
